# Evaluation of the effectiveness and safety of avapritinib in real-world Spanish cases with gastrointestinal stromal tumor and D842V-PDGFRA mutation

**DOI:** 10.1093/oncolo/oyaf062

**Published:** 2025-05-11

**Authors:** Isaac Nuñez Hernández, Cristina Gómez Palmero, Juan Ramón Delgado, Ana Nuño, Maria Ángeles Sala González, Ana González Ageitos, Héctor Aguilar, Pablo Ayala de Miguel, Elizabeth Condori, Roberto Díaz Beveridge, Jerónimo Martínez García, Gloria Marquina, Vanesa Varela-Pose, Joel Veas Rodríguez, César Serrano

**Affiliations:** Hospital Universitario Nuestra Señora de Candelaria, Santa Cruz de Tenerife, Ctra. Gral. del Rosario, 145, 38010, Spain; Fundación Investigación del cáncer en Canarias | FICIC, Av. Alcalde Díaz Saavedra Navarro, 31, 35001 Las Palmas de Gran Canaria, Las Palmas, Spain; Hospital Universitario de Toledo, Toledo, Av. del Río Guadiana, 45007, Spain; Hospital Universitario Vall d’Hebron, Pg. de la Vall d'Hebron, 119, Horta-Guinardó, 08035, Barcelona, Spain; Hospital Universitario Virgen de las Nieves (HUVN), Av. de las Fuerzas Armadas, 2, Beiro, 18014 Granada, Spain; Instituto de Investigación Biosanitaria ibs.GRANADA, Av. de Madrid, 15, Beiro, 18012 Granada, Spain; Hospital Obispo Polanco, Av. Ruiz Jarabo, s/n, 44002, Teruel, Spain; Hospital Universitario Basurto, Montevideo Etorb., 18, Basurtu-Zorrotza, 48013, Bilbao, Spain; Hospital Universitario de Toledo, Toledo, Av. del Río Guadiana, 45007, Spain; Fundación Instituto Valenciano de Oncología, Carrer del Professor Beltrán Báguena, 8, Campanar, 46009, Valencia, Spain; Complejo Hospitalario Universitario de Cáceres, Av. de la Universidad, 75, Norte, 10004 Cáceres, Spain; Hospital Santa Bárbara de Soria, Pº de Santa Bárbara, s/n, 42005, Soria, Spain; Hospital Universitari i Politècnic La Fe, Avinguda de Fernando Abril Martorell, 106, Quatre Carreres, 46026, Valencia, Spain; HCU Virgen de la Arrixaca, Ctra. Madrid-Cartagena, s/n, 30120 El Palmar, Murcia, Spain; Grupo Investigación en IMIB- Murcia, Ctra. Madrid-Cartagena, s/n, 30120 El Palmar, Spain; Department of Medical Oncology, Hospital Clínico San Carlos, Department of Medicine, School of Medicine, Universidad Complutense de Madrid (UCM), Instituto de Investigación Sanitaria (IdISSC), Calle del Prof Martín Lagos, S/N, Moncloa - Aravaca, 28040, Madrid, Spain; University Hospital of Santiago de Compostela (SERGAS), Rúa da Choupana, s/n, 15706, Santiago de Compostela, Spain; Translational Medical Oncology Group (Oncomet), Rúa da Choupana, s/n, 15706, Santiago de Compostela, Spain; Health Research Institute of Santiago de Compostela (IDIS), Rúa da Choupana, s/n, 15706, Santiago de Compostela, Spain; Hospital Arnau de Vilanova de Lleida, Av. Alcalde Rovira Roure, 80, 25198, Lleida, Spain; Musgrove Park Hospital, Somerset NHS Foundation Trust, Parkfield Dr, Taunton TA1 5DA, Reino Unido; Hospital Universitario Vall d’Hebron, Pg. de la Vall d'Hebron, 119, Horta-Guinardó, 08035, Barcelona, Spain; Vall d’Hebron Institute of Oncology (VHIO), Carrer de Natzaret 115-117, 08035 Barcelona, Spain

**Keywords:** gastrointestinal stromal tumor, GIST, *PDGFRA*, D842V mutation, avapritinib, treatment, outcomes

## Abstract

**Introduction:**

Gastrointestinal stromal tumors (GISTs) are the most common sarcoma subtype. Patients with unresectable or metastatic GISTs harboring the D842V mutation in the *PDGFRA* gene have a poor prognosis due to intrinsic resistance to imatinib and all other approved tyrosine kinase inhibitors. Avapritinib, targeting this mutation, is the first agent approved for patients with unresectable or metastatic GIST that have the PDGFRA D842V mutation. This study assesses the effectiveness and safety of avapritinib in real-world clinical scenarios involving Spanish patients with this mutation.

**Materials and methods:**

The AVARWE study is a descriptive, retrospective, multicenter observational study of 21 patients treated with avapritinib across 13 Spanish centers from June 9, 2023, to December 18, 2023. Data collected included patient demographics, disease characteristics, treatment history, and response rates based on RECIST criteria. The main outcomes, progression-free survival (PFS) and overall survival (OS), were measured, with safety assessed through adverse events documentation according to CTCAE criteria.

**Results:**

Median PFS 35.6 was months and median OS was 42.2 months, with survival rates at 1, 5, and 3 years demonstrating avapritinib effectiveness. The objective response rate was 76.2% for partial response and 4.8% for complete response. Avapritinib enabled surgical intervention in previously unresectable cases and was generally well-tolerated, with manageable adverse events.

**Conclusion:**

Avapritinib extends PFS and OS among patients with *PDGFRA* D842V-mutant GIST in real-world practice, mirroring pivotal trial outcomes. Its substantial activity supports its use as a first-line therapy for this subgroup. The manageable safety profile reinforces avapritinib viability for routine use. Given the rarity of these cases, it is advised to consult sarcoma-expert units.

## Graphical Abstract

**Graphical Abstract:**
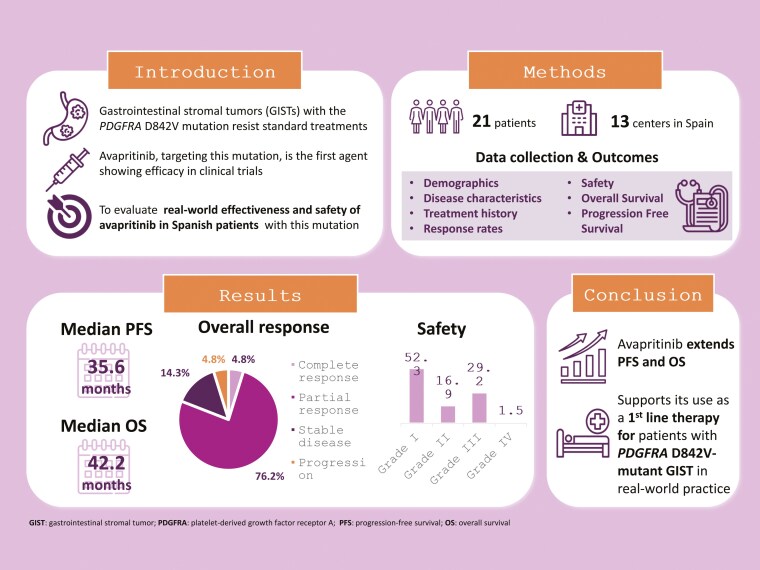

**Implications for practice**

This study underscores the effectiveness of avapritinib in treating patients with gastrointestinal stromal tumors (GIST) harboring the *PDGFRA* D842V mutation, a group traditionally facing limited treatment options and poor outcomes. Avapritinib offers unprecedented progression-free survival and overall survival and therefore can be considered as the first-line choice. Notably, the treatment has also facilitated surgical intervention for tumors previously deemed inoperable, thus providing a direct and impactful improvement in patient care and outcomes in the clinical management of GIST.

## Introduction

Gastrointestinal stromal tumors (GIST) are the most common malignant neoplasm of mesenchymal origin.^1^ Over 85% of GIST cases are driven by oncogenic mutations in the genes encoding the KIT receptor or platelet-derived growth factor receptor A (PDGFRA).^2^ These molecular alterations play a pivotal role in the treatment and clinical management of patients with advanced GIST, and thus most clinical guidelines recommend identification of these mutations through molecular diagnostic testing.^3,4^ Accordingly, various therapies tailored to GIST molecular subtypes are available.^5^ The European Medicines Agency (EMA) currently has approved 5 drugs for the treatment of advanced-stage GIST: imatinib, sunitinib, regorafenib, ripretinib, and avapritinib—all of which are tyrosine kinase inhibitors (TKIs). Specifically, avapritinib^6^ (formerly BLU-285, Blueprint Medicines Corporation), the only selective and potent inhibitor of mutant KIT and PDGFRA kinases, targeting D842V, has received approval for the treatment of patients with unresectable and metastatic GIST by both the United States Food and Drug Administration (FDA) (*PDGFRA* exon 18 mutation subpopulation) and the EMA (only in patients with the D842V mutation in exon 18 of *PDGFRA)*.^7,8^

The D842V mutation in the *PDGFRA* gene is detected in 5%-6% of GIST cases.^9^ Patients with unresectable or metastatic GIST harboring this mutation have a poor prognosis, with median progression-free survival (PFS) ranging from 2 to 10 months and median overall survival (OS) between 9 and 25 months for imatinib and other TKIs.^10-13^ This poor response is attributed to the primary resistance against all approved TKIs (all type II TKIs) prior to avapritinib, a type I kinase inhibitor.^14^

At present, efficacy data is primarily derived from the phase I NAVIGATOR clinical trial,^7,8^ with no evidence from routine clinical practice.^10^ In response to this lack of data, we conducted a retrospective observational study *AVApritinib Real-World Evidence* (AVARWE) on a cohort of patients treated with avapritinib that aimed to assess the effectiveness and safety of avapritinib in real-world scenarios involving Spanish patients diagnosed with GIST harboring the D842V-*PDGFRA* mutation.

## Materials and methods

### Study design and patients

The AVARWE study is a descriptive, retrospective, longitudinal, multicenter, observational study of a series of patients with advanced GIST treated with avapritinib in real-world clinical practice. Data from patients with unresectable/metastatic GISTs harboring a *PDGFRA* D842V mutation and on treatment with avapritinib were collected retrospectively from medical records, after obtaining the favorable opinion of the Vall De Hebron Hospital ethics committee (Barcelona) from June 9, 2023, to December 18, 2023. An informed consent form and an information sheet were provided to all living patients at the time data collection began. For cases where obtaining informed consent was not feasible, an exemption was granted by the Ethics Committee in compliance with the International Society for Pharmacoepidemiology Guidelines for Good Pharmacoepidemiology Practices for observational studies and in accordance with Good Clinical Practice guidelines, as well as applicable local laws and regulations.^15,16^ Inclusion criteria were: (1) histologic diagnosis of GIST with the D842V mutation in the *PDGFRA* gene, (2) aged 18 years or older, (3) locally advanced or metastatic disease confirmed by radiological imaging techniques, (3) treated for locally advanced or metastatic disease with avapritinib in any line of treatment, (5) treated in the participating centers with avapritinib under compassionate or extended use or as foreign medication, and (6) signed informed consent to participate in the study, unless exempted as described above. Patients treated with avapritinib exclusively within the context of the NCT03465722 clinical trial were excluded.

### Treatments and response to treatment

Data were collected on the treatment with avapritinib, as well as previous therapies. Up to 6 lines of prior treatment were documented for each patient, incorporating data on treatment responses, dates of best response, and the dates of radiological progression for each line of therapy. All radiological evaluations were performed every 8–12 weeks, following institutional guidelines. The objective response rate (ORR) was collected retrospectively from the medical records as determined by each investigator during routine clinical practice. Response to drug was evaluated by each investigator at the time of scan assessment according to clinical practice standards, with an approximation to Response Evaluation Criteria in Solid Tumors version 1.1(RECIST v1.1) criteria,^17^ categorizing it as follows: complete response, partial response, stable disease, and disease progression.

### Safety analysis

All adverse events (AEs) occurring during the treatment with avapritinib in clinical practice were collected by the primary oncology team and documented in electronic medical records. The type and severity of each event were classified according to the criteria outlined in the latest version (V. 5.0.) of the Common Terminology Criteria for Adverse Events (CTCAE)^18^ All AEs were retrospectively collected from medical records for this study.

### Exploratory analysis: estimation of growth modulation index

The growth modulation index (GMI) is the ratio of time to progression with the *nth* line (TTPn) of therapy to TTPn-1 with respect to the (n-1)st line.^19^ We explored the interest threshold: GMI > 1.33. GMI > 1.33 was defined by Von Hoff as a sign of drug activity.^20^ The GMI estimation was estimated to calculate the PFS ratio and OS ratio of avapritinib treatment compared to the first-line treatment. The GMI could be estimated for 12 cases who had previous treatment with other TKI. The GMI was categorized for the analysis into 2 groups: Group A: defined as non-responders and consisting of patients whose GMI or survival time with avapritinib/survival time with first-line treatment was < 1.33 and Group B: defined as responders and consisting of patients whose GMI or survival time with avapritinib/survival time with first-line treatment was ≥1.33. After categorization, groups A and B were compared with respect to PFS and OS.

### Outcomes

Baseline characteristics of patients and disease specifics, along with anatomopathological features of the tumors, were gathered. Data on previous treatments with TKIs were also documented. Additionally, details on avapritinib treatment including information regarding the frequency, type, and severity of AEs, dose reductions, interruptions, and treatment discontinuations, as well as patient responses and status were collected retrospectively from medical records. Patients could experience more than one AE and up to a maximum of 6 AEs per patient were collected. All data were retrospectively collected from medical records.

### Statistical analysis

Baseline demographics, clinical variables, and safety analysis were summarized as median and interquartile range (IQR), or means and standard deviation (SD), and frequency data (proportion), as appropriate. PFS was calculated from the beginning of avapritinib treatment until the date of progression according to *RECIST criteria v1.1*. OS was calculated from the beginning of avapritinib treatment until the date of death or end of follow-up. For PFS and OS, univariate survival analysis was performed, survival curves were constructed using the Kaplan-Meier method, and median PFS and OS and their 95% confidence intervals (CIs) were estimated. GMI was calculated for PFS and for OS. Survival analysis was performed to calculate the GMI and PFS ratio and OS ratio of avapritinib treatment compared to the first-line treatment. The survival probability and differences between GMI groups were estimated using the Kaplan-Meier method and the Cox model. The Kaplan-Meier curves of each line were compared using the log-rank test. In the Cox model, hazard ratios (HRs) and 95% CI were calculated. Analyses were performed using R statistical software (version 4.1.1, R Bioconductor).

This study was reported in accordance with the Strengthening the Reporting of Observational Studies in Epidemiology (STROBE) statement. A checklist of the STROBE statement for cohort studies is shown in Supplementary Table S1.

## Results

### Characteristics of the real-life study population

Patient data was collected from June 9, 2023, to December 18, 2023. Patients included were treated with avapritinib from January 5, 2018, to December 18, 2023, the date of cutoff and end of data collection. In total, 21 patients from 13 Spanish centers participating in the AVARWE study were analyzed (Table 1). At the data cut-off (December 18, 2023) median follow-up was 26.2 months.

**Table 1.** Demographic and clinical characteristics of the AVARWE population.

Characteristics	Overall (*N* = 21) N (21)
Gender, *N* (%)	
Male	12 (57.1%)
Female	9 (42.9%)

Age at diagnosis	
Median, IQR [Q1, Q3]	60.3 (51.6-64.1)
Range (min, max)	(25.2-68.0)

Age at avapritinib initiation	
Median, IQR [Q1, Q3]	62.6 (53.8-66.6)
Range (min-max)	(40.6-71.6)

Region *N*, (%)	
Andalucía	2 (9.5 %)
Aragón	2 (9.5 %)
Canary Islands	4 (19.0 %)
Castilla La Mancha	1 (4.8 %)
Castilla and León	1 (4.8 %)
Catalonia	3 (14.2%)
Madrid region	1 (4.8 %)
Valencia region	2 (9.5 %)
Extremadura	1 (4.8 %)
Galicia	1 (4.8 %)
Basque Country	2 (9.5 %)
Murcia region	1 (4.8 %)

ECOG	
0	11 (52.4 %)
1	9 (42.9 %)
3	1 (4.8 %)

Comorbidities Cardiac Hepatic Other neoplasm Other none Comorbilidades	
Cardiac	1 (4.8 %)
Hepatic	1 (4.8 %)
Other neoplasm	8 (38.1%)
Other	6 (28.6 %)
None	5 (23.8 %)

Abbreviations: ECOG, Eastern Cooperative Oncology Group; IQR, interquartile range; *N*: number of patients.

The median age (range) at diagnosis of GIST and at avapritinib initiation was 60.3 years (25.2-68.0) and 62.6 years(40.6-71.6), respectively. Interestingly, 38.1% of the patients had another neoplasm in addition to GIST (concomitant and/or previous). At the time of initiation of avapritinib, the majority of the patients were in good condition, with 52.4% having normal performance status (Eastern Cooperative Oncology Group [ECOG] = 0), and 42.9% an ECOG of 1 (Table 1).

Eighty five percent of primary tumors were located in the stomach, while 4.5% were in the small bowel and 9.5% in other locations (Table 2). At the time of diagnosis, 52.4% of patients presented with localized disease, 14.3% with locally advanced disease (unresectable but not metastatic), and 33.3% with distant metastases. Among the cases that presented metastases (*N* = 20), 55% were found in the liver and 45% in the peritoneum. Regarding histology, 42.9% of tumors presented epithelioid characteristics, 23.8% were spindle cell tumors, and 23.8% were mixed histology. In primary tumor biopsies, the median number of mitoses per mm^2^ was 7 (IQR 3-14), and the median localized tumor size was 16 cm (IQR 8.82-24.6).

**Table 2.** Characteristics of the disease in the AVARWE population.

Variable	*N* = 21
Tumor histology	
Epithelioid	9 (42.9%)
Spindle Cell	5 (23.8%)
Mixed	5 (23.8%)
Unknown	2 (9.5%)

Primary tumor location	
Stomach	18 (85.7%)
Intestine	1 (4.8%)
Other	2 (9.5%)

Disease at diagnosis	
Localized	11 (52.4%)
Locally advanced	3 (14.3%)
Metastatic	7 (33.3%)
**Metastatic relapse (if localized disease)**	*N* = 11
Yes	11 (100%)
No	0 (0%)
**Metastatic relapse (if locally advanced disease)**	*N* = 3
Yes	2 (67.7%)
No	1 (33.3%)
**Metastatic relapse (if metastatic disease)**	*N* = 7
Yes	1 (14.3%)
No	6 (85.7%)
**Metastatic relapse (overall)**	*N* = 21
Yes	14 (66.6%)
No	7 (33.3%)
**Metastatic disease onset (avapritinib)**	*N* = 21
Yes	20 (95.2%)
No	1 (4.8%)
**Metastasis location**	*N* = 21
Liver	11 (55.0%)
Peritoneum	9 (45%)
**Number of mitosis × 5 mm** ^**2**^	*N* = 20
Shapiro-Wilk *P*value	<.001
Median (IQR)	7 (3-14)
Mean (SD)	12 (15.5)
**Tumor size (local) (cm)**	*N* = 10
Shapiro-Wilk p Value	0.224
Median (IQR)	16 (8.82-24.6)
Mean (SD)	16.2 (9.1)

Abbreviations: IQR, interquartile range; *N*, number of patients; SD: standard deviation.

### Description of previous treatments

In total, 57.1% of patients had received at least one prior line of therapy before initiating treatment with avapritinib, while 42.9% (9/21) were TKI-naïve. Among the patients who had received prior treatments (*N* = 12), the most common first-line therapy was imatinib, accounting for 91.7% of cases, and crenolanib in 8.3%.

The median duration of the first line of treatment was 4.96 months (IQR 2.52-10.2), thus confirming the limited benefit of imatinib in this setting. The best response to this initial treatment was tumor progression in 50% of cases, and stable disease in the remaining population.

### Activity of avapritinib in locally-advanced and metastatic patients

**Figure 1.**
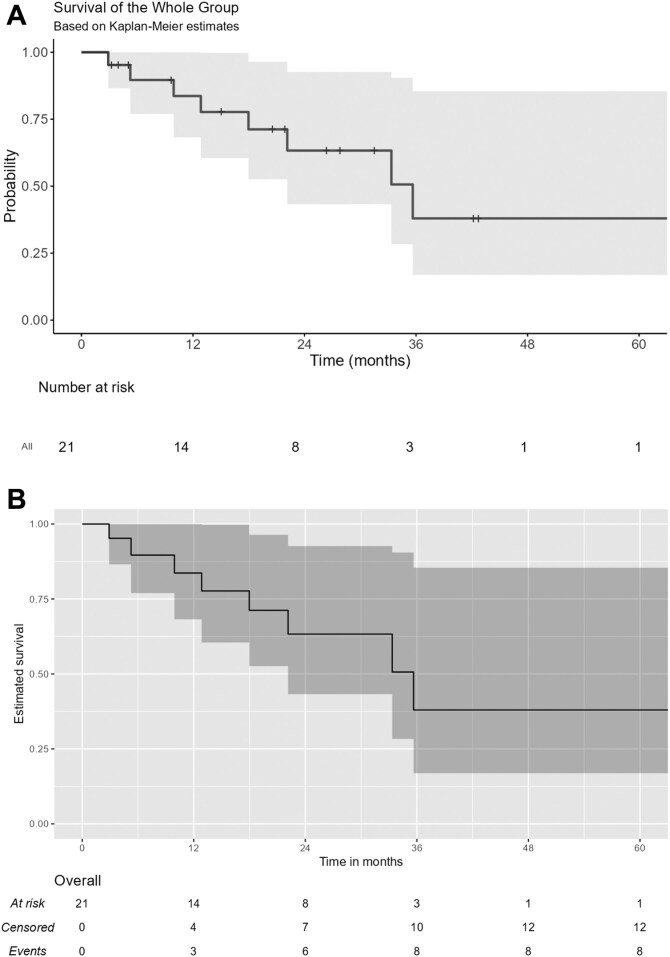
Kaplan-Meier curves for progression-free survival (PFS) with avapritinib in the AVARWE population. (A) Kaplan-Meier curve for PFS with avapritinib in the AVARWE population. (B) KMunicate-Style Kaplan-Meier curve for PFS with avapritinib in the AVARWE population.

**Figure 2.**
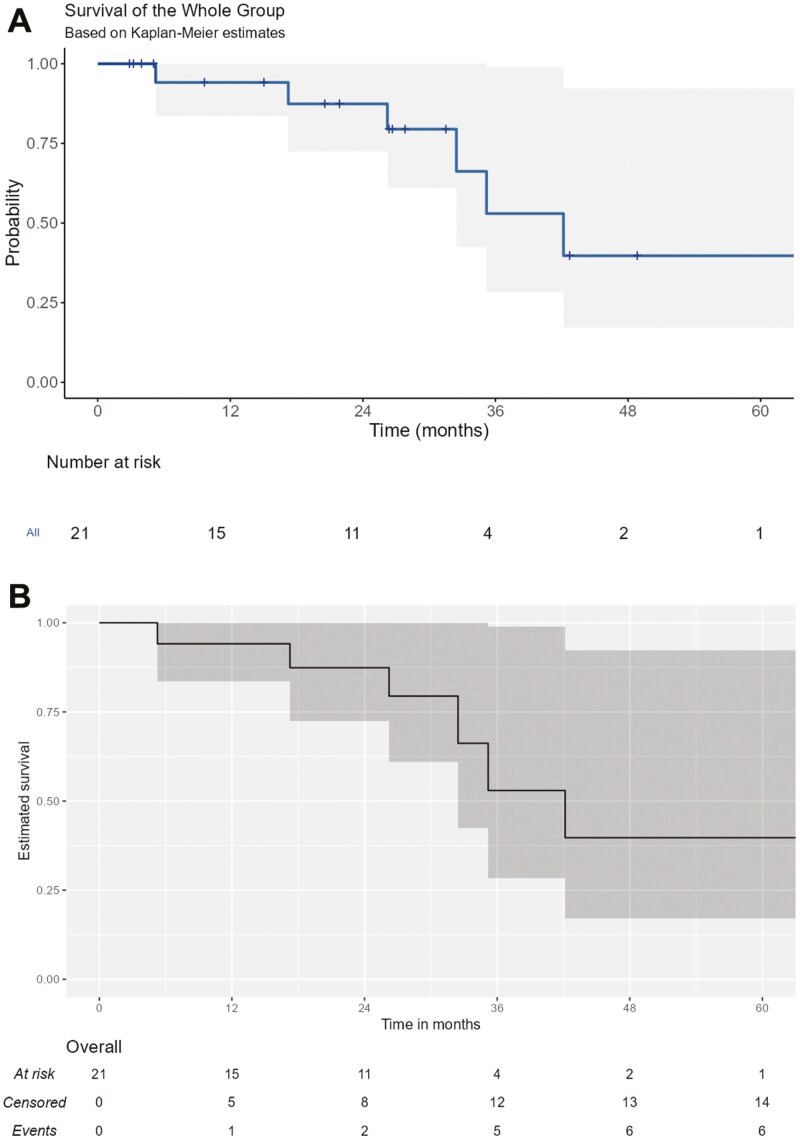
Kaplan-Meier curves for overall survival (OS) with avapritinib in the AVARWE population. (A) Kaplan Meier curve for OS with avapritinib in the AVARWE population. (B) Kaplan Meier curve for OS with avapritinib in the AVARWE population.

At the data cutoff date (December 18, 2023), the median follow-up from avapritinib initiation was 26.2 months. The median PFS (95%CI) for the AVARWE population after avapritinib initiation was 35.6 months (22.1—not reached [NR]) (Figure 1A and 1B). The projected PFS rates at 1, 3, and 5 years were 84% (95% CI: 68–100), 38% (95% CI: 17–85), and 38% (95% CI: 17–85), respectively. The median OS (95%CI) was at 42.2 months (32.5-NR) (Figure 2A and 2B). The projected OS rates at 1, 3, and 5 years were 94% (95% CI: 84–100), 53% (95% CI: 28–98), and 40% (95% CI: 17–92.3), respectively.

Of 21 patients evaluable for response, the overall response rate for avapritinib treatment according to RECIST criteria, was 81% (*N *= 17), with complete response seen in 4.8% (*N* = 1) of the patients and partial response in 76.2% (*N* = 16). Stable disease was observed in 14.3% (*N* = 3), and only 4.8% (*N *= 1) of patients experienced disease progression at the first radiological evaluation. Taken together, the clinical benefit rate (sum of complete and partial response, and stable disease) of avapritinib in this real-world population was 95.2% (*N* = 20).

### Real-world management of avapritinib therapy

#### Tumor shrinkage and surgical procedure after treatment with avapritinib

**Figure 3.**
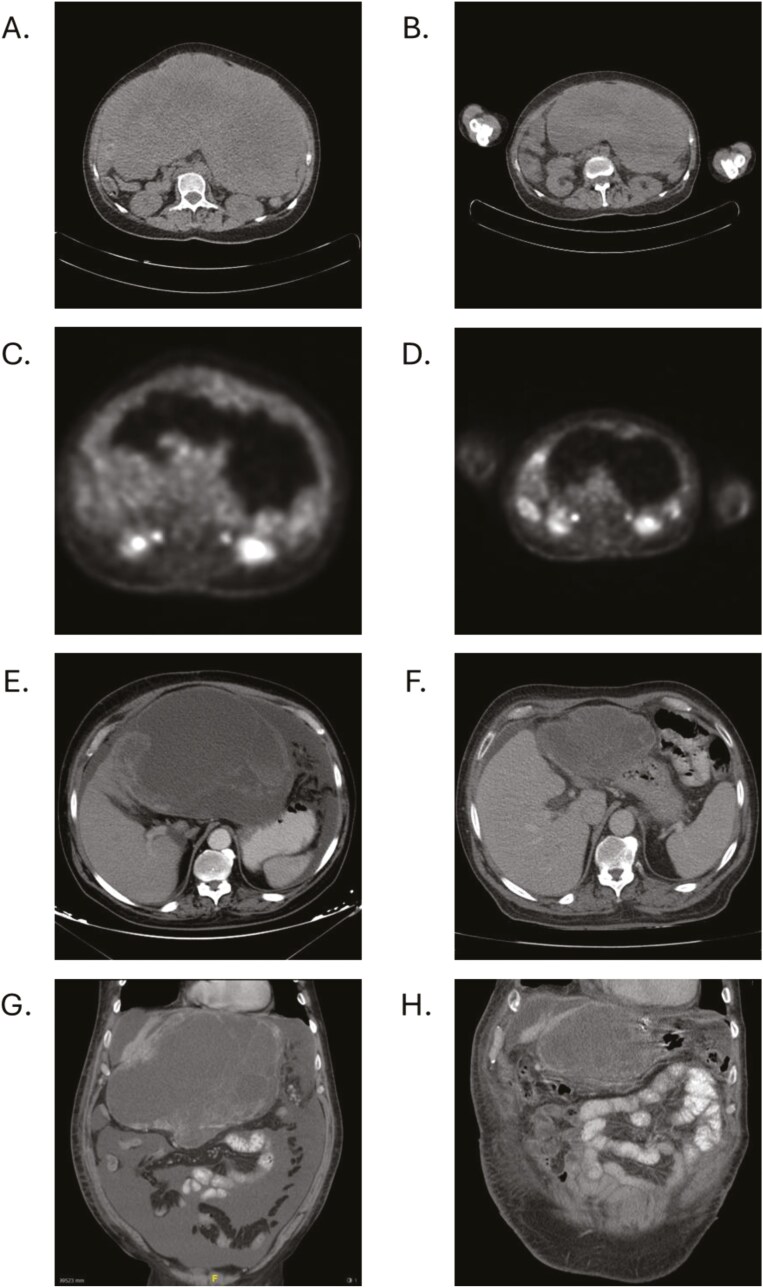
Surgery after treatment with avapritinib in 2 patients. Figure 3A and C: Pre-treatment with avapritinib: large abdominopelvic mass with hypometabolic areas due to necrosis occupying the epigastrium-mesogastrium and left iliac fossa of similar size, 15.8 cm  × 27cm with diffuse mild metabolic enhancement. Left subphrenic nodular lesions adjacent to colon, adjacent to stomach, and right perihepatic region. Figure 3B and D: Post-treatment with avapritinib: large abdominopelvic mass with hypometabolic areas due to necrosis and calcifications, smaller than in the previous study, occupying the mesogastrium-hypogastrium and left iliac fossa, approx. 11.8 cm  × 21cm with the slight peripheral metabolic increase attributable to partial response. Discrete reticulation of the left subphrenic omental fat, less significant than in the previous study, as well as discrete perisplenic and pelvic ascites without clear pathological foci. Figure 3E and G Pre-treatment with avapritinib showing a large abdominal mass in axial (3E) and coronal (3G section). Figure 3F and H Post-treatment with avapritinib showing a major partial response in axial (3F) and coronal (3H) sections that allowed surgical resection.

In 2 patients, avapritinib treatment enabled surgical removal of primary tumors that were initially deemed unresectable. In one case where surgery was feasible, the patient initially presented with a large abdominopelvic mass measuring 15.8 cm  × 27 cm, characterized by hypometabolic areas due to necrosis and calcifications (Figures 3A and 3C). After only 4 months of avapritinib, the mass had reduced by 22%, measuring 11.8 cm  × 21 cm (Figure 3B and D). Further tumor reduction led to a sustained partial response. After 16 months of treatment, the largest mass could be resected and small peritoneal lesions were fulgurated, becoming an R0 surgery. In a second case, the primary tumor had a maximum diameter size of 25 cm and was accompanied by peritoneal implants and ascites (Figure 3E and G). After 13 months of avapritinib treatment, a subtotal gastrectomy and hepatectomy were performed, and observing that the peritoneal implants were absent (Figure 3F and H). Avapritinib was continued after the patients were discharged due to the metastatic nature of their disease. Together, pre-treatment with avapritinib may allow debulking surgery of large tumor masses and concomitant metastases in selected patients.

#### Safety analysis

Regarding the safety and tolerability of avapritinib, 85.7% of patients experienced at least one adverse event (AE). During the treatment period, a total of 65 AEs were documented (Table 3), only one of them grade IV and none of them grade V. Of the recorded AEs, 34 (52.3%) were classified as grade I, 11 (16.9%) as grade II, 19 (29.2.%) as grade III and 1(1.5%) as grade IV.

**Table 3.** Safety analysis.

Types of adverse events, *N* (%)	Overall (*N* = 65)	Grades I and II (*N* = 45)	Grades III and IV (*N* = 20)
**Hematological AEs**	17 (26.2%)	7 (15.6%)	10 (50%)
**Non-hematological AEs**	48 (73.8%)	39 (84.4%)	10 (50%)
**Non-hematological AEs**	48 (73.8%)	39 (84.4%)	10 (50%)
Gastrointestinal AEs	16 (24.6%)	14 (31.1%)	2 (10.0%)
Neurological AEs	13 (20.0%)	7(15.6%)	6(30.0%)
Facial edema	6(9.2%)	6(13.3%)	0 (0.0%)
Hepatic AEs	5(7.7%)	4(8.9%)	1(5.0%)
Renal AEs	4 (6.2%)	3 (6.7%)	1(5.0%)
Asthenia	3 (4.6%)	3 (6.7%)	0 (0.0%)
Extremity edema	1 (1.5%)	1 (2.2%)	0 (0.0%)
**Hematological AEs**	17 (26.2%)	7 (15.6%)	10 (50%)
Anemia	7 (10.8%)	3 (6.7%)	4 (20%)
Neutropenia	5 (7.7%)	1 (2.2%)	4 (20%)
Lymphopenia	3 (4.6%)	2 (4.4%)	1 (5%)
Thrombocytopenia	2 (3.1%)	1 (2.2%)	1 (5%)

Abbreviation: *N*, number of patients.

In the AVARWE population, most AEs were of a hematological nature, accounting for 26.2% of all AEs, with anemia being the predominant hematological toxicity. This was followed by gastrointestinal and neurological AEs, which constituted 24.6% and 20% of the total AEs, respectively (Table 3). Grade III AEs were neurological AEs (6/19), neutropenia (4/19), anemia (3/19), gastrointestinal AEs (2/19), renal AEs (1/19), thrombopenia (1/19), lymphopenia (1/19) and hepatic AEs (1/19). Four (21%) of these neurological grade III AEs (cognitive impairment) (4/19) were conducted to the definitive discontinuation of treatment. One grade IV AE (anemia) required hospitalization and transfusion, eventually leading to a reduction in the avapritinib dose.

#### Dose reductions, interruptions, and withdrawals

When initiating treatment with avapritinib, all but 3 patients started with an initial dose of 300 mg. Treatment initiation with a lower dose was justified by age, frailty, or the combination of both. Additionally, a significant proportion of patients, approximately 66.7% (14/21) patients, required at least one dose reduction during the course of treatment with avapritinib, while 81% (17/21) had some form of treatment interruption.

The most frequent reasons for dose reductions and interruptions included grade III AEs such as hepatic, gastrointestinal, neurological (cognitive impairment) and hematological toxicity (anemia and neutropenia) and other toxicities and complications such as perianastomotic ulcers, multiple punctate cerebral hemorrhage, palpebral edema, or Gilbert syndrome.

In 4 patients (19.1%), treatment was definitively discontinued due to neurological toxicities including severe cognitive impairment. One of them was diagnosed with dementia with Lewy bodies and in another, choreic movements of the trunk appeared after surgery with attentional memory impairment and bradykinetic syndrome. The median age of these 4 patients was 62.9 (range, 62.2-68.5) years. None of these patients had predisposing conditions for cognitive impairment. All patients had 300 mg QD as the starting dose and 3 patients had dose reductions and interruptions before definitive treatment withdrawal.

### Growth modulatory index for PFS and OS

Of the total population, the GMI could be calculated in 12/21 patients who had received at least one previous line of treatment. These 12 patients had received a median of 1.5 therapies (range 1-6) prior to the initiation of avapritinib. The GMI of avapritinib treatment for PFS and OS was calculated with respect to the time on the first-line therapy, which consisted of imatinib in 11 cases and crenolanib in one other patient. Ten out of 12 patients had a GMI above the 1.33 threshold, clearly indicating the benefit of avapritinib in comparison with first-line imatinib in the majority of the patients.

PFS and OS were further investigated in Group A (GMI < 1.33, indicating a ratio in favor of first-line treatment) and Group B (GMI ≥ 1.33, indicating a ratio in favor of avapritinib treatment). PFS (95%CI) was 9.92 months (9.92—NR) in Group A and 35.65 months (33.35—NR) in Group B, a difference that was not statistically significant (HR, 1.04; 95% CI, 0.11-9.72, *P = *.971). Likewise, no differences were observed either regarding OS, with a median OS of 29.7 months (17.2—NA) in Group A and not reached in Group B (35.2- NR), thus not reaching the statistical significance (HR, 0.39; 95% CI, 0.06-2.41; *P* = .310). Taken together, although the length of the follow-up may have an impact on these results, avapritinib treatment in metastatic *PDGFRA* D842V-mutant GIST patients appears to improve the outcomes to a greater extent than first-line treatment.

## Discussion

Before the regulatory approval of avapritinib, the outcomes of patients with advanced or metastatic *PDGFRA* D842V-mutant GIST were dismal due to the intrinsic resistance to the TKIs commonly used in GIST therapeutics.^10-13^ Avapritinib, a type I TKI targeting specifically the activation loop of *PDGFRA*, is the first-ever treatment to show activity against this rare GIST molecular subtype. The regulatory approval came after the unprecedented results were observed in 56 *PDGFRA* D842V-mutant GIST patients in the phase I NAVIGATOR clinical trial.^7,8^ Given the limited number of patients included in the trial—due to the rarity of this molecular subgroup—as well as the well-known restrictive context of clinical trials, we sought to provide real-world evidence for effectiveness, safety, and clinical management in real-world conditions.^21^ This is, to our knowledge, the first study on real-life treatment of GIST patients with avapritinib.

Our real-world findings first confirm the substantial improvement obtained with avapritinib in any line of treatment, with figures following a similar trend to those from the patients treated in the NAVIGATOR trial. Herein, as in the clinical trial, the majority of the patients achieved a significant tumor shrinkage (overall response rate of 81%) and disease control at the first radiological evaluation (clinical benefit rate of 95.2%). The slightly higher ORR reported in the pivotal trials (95%) may be due to numerical differences related to the low number of patients, the real-world management of avapritinib, or both. However, the outcomes (PFS and OS) in our AVARWE cohort follow a similar trend to those reported in the long-term efficacy analyses of the NAVIGATOR clinical trial.^8^ Indeed, considering a similar median long-term follow-up (26.2 months in the AVARWE population and 27.5 months in the NAVIGATOR population).^7,8^ These results also showed a similar tendency with the subgroup analysis for PFS performed in the VOYAGER phase III clinical trial for patients carrying the *PDGFRA* D842V mutation.^22^ Therefore and collectively, PDGFRA D842V mutation GIST patients treated with avapritinib in real-life conditions achieve similar clinical benefit and improvement in survival and response outcomes as those reported in scientific literature.^22^

Notably, avapritinib treatment enabled debulking surgery in 2 patients with an extent of disease deemed unresectable prior to treatment initiation. Extensive surgeries, including debulking procedures, after first-line imatinib therapy have been shown to extend OS in selected *KIT/PDGFRA*-mutant GIST patients with locally advanced (unresectable at diagnosis but not metastatic) and/or metastatic disease.^23,24^ Therefore, it is conceivable that, given the high response rate achieved with avapritinib in *PDGFRA* D842V-mutant GISTs, these types of surgical procedures may also be feasible in this subset of patients and obtain similar positive outcomes thus potentially expanding the survival in this population. This decision should be always discussed individually with the patient and within a multidisciplinary tumor board and preferably performed in a sarcoma-expert institution. Avapritinib treatment was continued in both cases given the metastatic nature of their disease. Further studies are needed to determine the optimal duration of avapritinib therapy in the preoperative and postoperative settings.^25^

All patients but 3 started at the standard dose of avapritinib 300 mg daily and the majority of them (85.7%) of patients experienced at least one AE. AEs were overall manageable, being 52.3% grade 1, 16.9% grade 2, 29.2% grade 3 and 1.5% grade 4. Although the rates of dose reduction (67% vs 45%) and dose interruptions (81% vs 66%) were slightly higher in this series than those observed in the phase I clinical trial,^7,8,26^ the proportion of treatment discontinuation due to AEs was similar between both groups (19% vs 17%). The most frequent AEs reported in our cohort were hematological (26.2%), gastrointestinal (24.6%) and neurological (20%). This distribution and frequency of toxicities are likely explained by underreporting toxicities due to the retrospective nature of this study. Additionally, discrepancies often exist between clinical trial results, where populations are highly selected, and real-world outcomes, where patients typically have more comorbidities and poorer general health.^21,27,28^

Of note, the incidence of grade 3 neurological AEs seemed higher here than in the original clinical trial, being all these AEs of cognitive nature, which raises importance as those led to the permanent discontinuation of avapritinib treatment in 4 patients. However, the low number of patients in this retrospective study and its inherent limitations do not allow any direct comparisons with any of the clinical trials performed in this indication. Cognitive effects occur with avapritinib more commonly than with other TKIs used in the treatment of GIST, and specific management guidelines have been published to ameliorate its occurrence.^26^ Since GIST patients can be treated outside sarcoma-referral institutions, and patients with GIST *PDGFRA* D842V constitute a very rare population, it is critical to stress the education of community oncologists in the careful management of neurological toxicities which includes specific questions at each visit, close monitoring during the first months, considering lower doses in aged patients upfront, timely dose adjustments (interruptions and/or reductions), and consultation with GIST-expert oncologists.

This study is subject to the inherent limitations of a retrospective, longitudinal observational design. Despite these limitations, the robustness of the analysis is enhanced by the completeness of the data for most variables, including all study endpoints, which minimizes the potential biases typically associated with missing data. Selection bias was also mitigated by including all Spanish patients treated with avapritinib outside of clinical trials up to the study’s closing date, providing a comprehensive overview of real-world outcomes. However, a notable limitation of the study is the sample size, which may have been insufficient to detect significant associations in the exploratory analysis of the GMI. Avapritinib has been approved by the FDA and EMA in any line of treatment.^7,8^ However, the unprecedented activity in the D842V *PDGFRA*-mutant subpopulation supports its use earlier in the clinical course to maximize its clinical benefit. Indeed, in this real-world study, the treating physicians decided to initiate avapritinib in the first-line setting in 9 patients, while the remaining 12 patients with longer treatment histories had received between 1 and 6 previous lines before avapritinib. Beyond the improvement in PFS and OS achieved by avapritinib in a real-world setting, the GMI in the previously pre-treated population clearly shows a benefit in the outcomes derived from avapritinib in comparison to first-line imatinib. Therefore, these results from this cohort support the use of avapritinib as early as possible during the clinical history of these patients, albeit acknowledging the limited prospective data available.

## Conclusion

This real-world study, conducted in 13 different institutions across Spain confirms the unprecedented activity demonstrated by avapritinib in the NAVIGATOR clinical trial, which led to the regulatory approval of this agent in *PDGFRA* D842V-mutant GIST. These data support its early application in the clinical management of these patients. Additionally, the high rate of responses seen with avapritinib combined with the lack of other effective treatment options underscores the importance of individualizing the incorporation of local treatment options to maximize the benefit of this agent. This should be done preferably in sarcoma-expert institutions. Finally, while the toxicities were generally manageable, a note of caution should be taken regarding the cognitive effects seen at higher strengths in order to maintain avapritinib treatment while preserving the quality of life of these patients thus early identification and dose reduction are critical in managing these AEs.

## Supplementary Material

oyaf062_suppl_Supplementary_Tables_1

## Data Availability

The data underlying this article will be shared on reasonable request to the corresponding author.
